# Using simulated bronchoscopy to improve training on intensive care units in a north London NHS trust

**DOI:** 10.1016/j.fhj.2024.100186

**Published:** 2024-09-12

**Authors:** Ewan Christopher Mackay, Lina Grauslyte

**Affiliations:** a King's College Hospital NHS Trust, Denmark Hill, London SE5 9RS, UK; b Whipps Cross University Hospital, London E11 1NR, UK

**Keywords:** Bronchoscopy, Simulation, Curriculum, Education

## Abstract

Flexible bronchoscopy is a vital tool that is used for both diagnostic and therapeutic indications on the intensive care unit. During the COVID pandemic, training opportunities to perform such a skill were decreased and novel methods of teaching, including using bronchoscopy simulators, were explored out of necessity. The aim of this quality improvement project was to investigate how training on an intensive care unit prepared trainees for carrying out bronchoscopy, and whether a simulated bronchoscopy session with a high-fidelity simulator could be a useful adjunct to gain experience. Our pilot, although limited in patient numbers, suggested that current teaching methods might not be adequate for building trainee confidence and that a session with a bronchoscopy simulator could serve as a remedy to this with a significant increase in self-reported confidence. Moving forwards in a post-pandemic era, this serves as a lesson learnt from COVID and would benefit from further development in this area.

## Introduction

Flexible bronchoscopy is a procedure that is often encountered on the intensive care unit, for both diagnostic and therapeutic purposes.^1^^,^^2^ Common indications include (but are not limited to) difficult intubation, obtaining bronchial washings to aid in guiding antimicrobial management, removing foreign bodies, and for removing mucus plugs to improve ventilation.^2^ Knowledge of the indications and endobronchial anatomy is fundamental to being able to perform the investigation appropriately and to minimise complications.^1^ However, due to the COVID pandemic and concerns over the aerosolising nature of the procedure and high PPE requirements,^3^ opportunities for trainees to perform this valuable skill have significantly decreased, and it has been suggested that this may have a dramatic impact on trainee aptitude and confidence in performing bronchoscopy and more advanced sampling techniques.^4^

A number of bronchoscopy simulators have been put forward as tools to help with training, varying from low to high fidelity,^5^^,^^6^ which are discussed at length in our discussion section. Literature has previously suggested that they can be very useful as training tools for increasing technical ability,^7^^,^^8^ although there is still a lack of comparative evidence for superiority between using computerised simulators over more simple anatomical models.^8^ Regardless, simulation has demonstrated itself to be an effective means of training and could offer benefits over the current apprenticeship-based model that is widely adopted.^9^ Simulation also has the added benefit of being able to train personnel without risk of direct harm to patients, and can be cost-effective, minimising expensive procedure room time.^7^

With the growing need for more doctors and a lack of learning opportunities exacerbated during the COVID pandemic, the use of bronchoscopy simulators may become increasingly recognised in future postgraduate medical training.^10^ The aim of this quality improvement project was to investigate how well postgraduate training programmes currently prepared trainees on intensive care for carrying out bronchoscopy, and whether a simulated bronchoscopy session with a high-fidelity simulator could be a useful adjunct to gain experience in performing this highly useful skill.

## Methods

Trainees were recruited for the project from the intensive care units of two large north London teaching hospitals. Trainee numbers per session varied from two to six, with 20 participants recruited in total, with all included in the final analysis. The session had a short didactic learning component covering anatomy and indications for bronchoscopy, but the main body of the session (please see Fig. 4) was dedicated to trainees navigating the bronchial tree with tutoring in real-time from a senior higher-grade anaesthetist, as well as a respiratory registrar.

The ORSIM bronchoscopy simulator (Airway Simulation Limited, Auckland, New Zealand) was used, which consists of a screen, a mock endoscope and an interface to feed the endoscope through (please see Fig. 3) to accurately simulate a real flexible bronchoscopy.^5^ Interactive cases were used to highlight learning points, and each case had variables that could be changed such as entry point (nasal or oral), position and use of oxygen, with trainees selecting how they would proceed given the intricacies of the case. The endoscopic images were projected onto a large screen, so that the trainees could navigate the upper/lower airways while other participants could also watch how the scenario unfolded.

The teaching session was roughly split into two parts with the first focusing on upper airway emergencies, while the latter part focused on endobronchial anatomy, naming bronchopulmonary segments and navigating the lower airways.

Questionnaires were used both pre- and post-intervention and asked questions relating to the candidates’ previous experiences of bronchoscopy, number of bronchoscopies performed and their confidence navigating the anatomy of the bronchial tree (see Fig. 5 for an example of such a questionnaire). Paired *t*-tests were used to assess for clinical significance and correlations. Free text questions were also included. These were included in the analysis below.

## Results

**Fig. 1:**
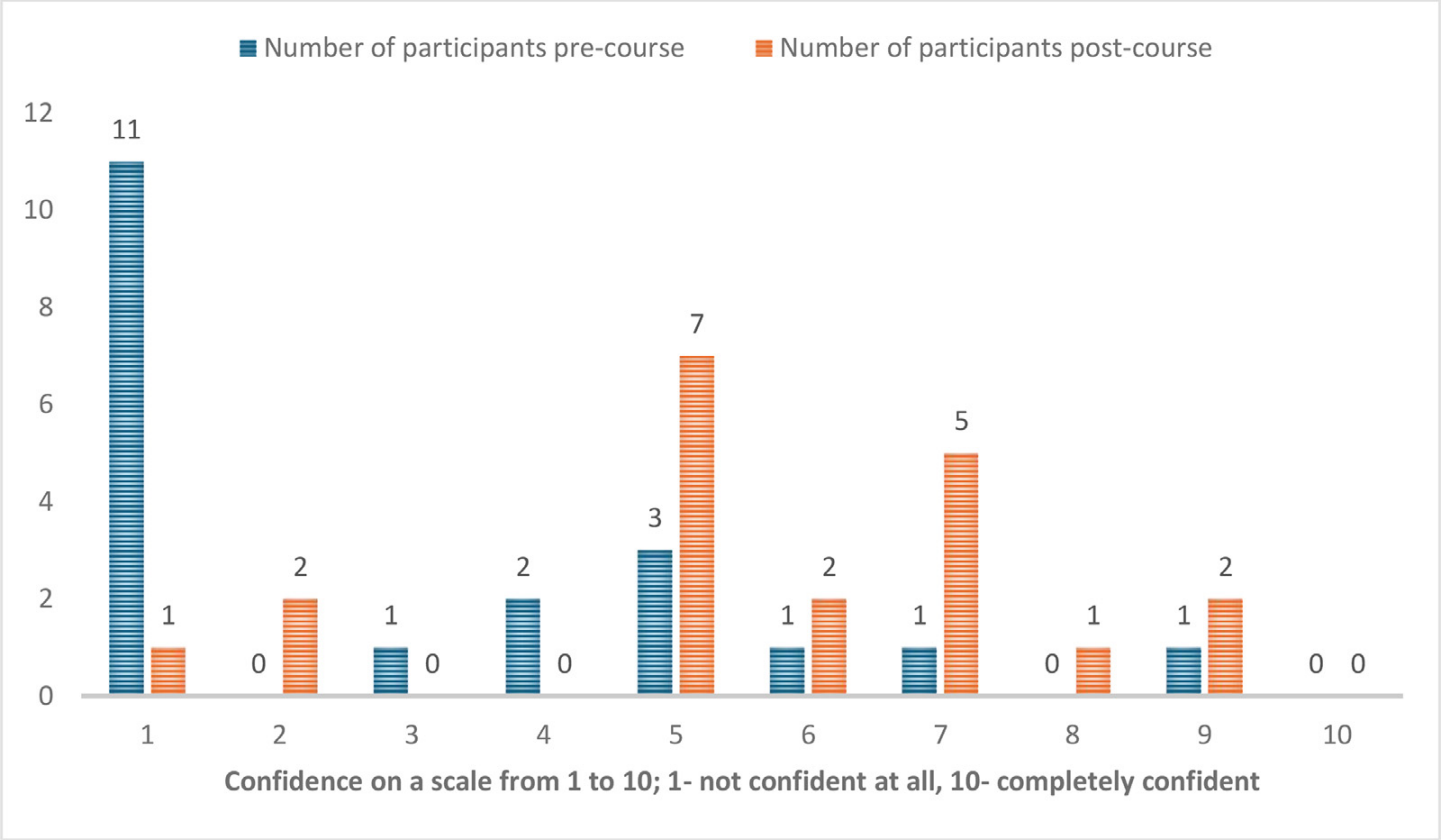
Self-reported confidence to perform a bronchoscopy unsupervised pre- and post-course.

**Fig. 2:**
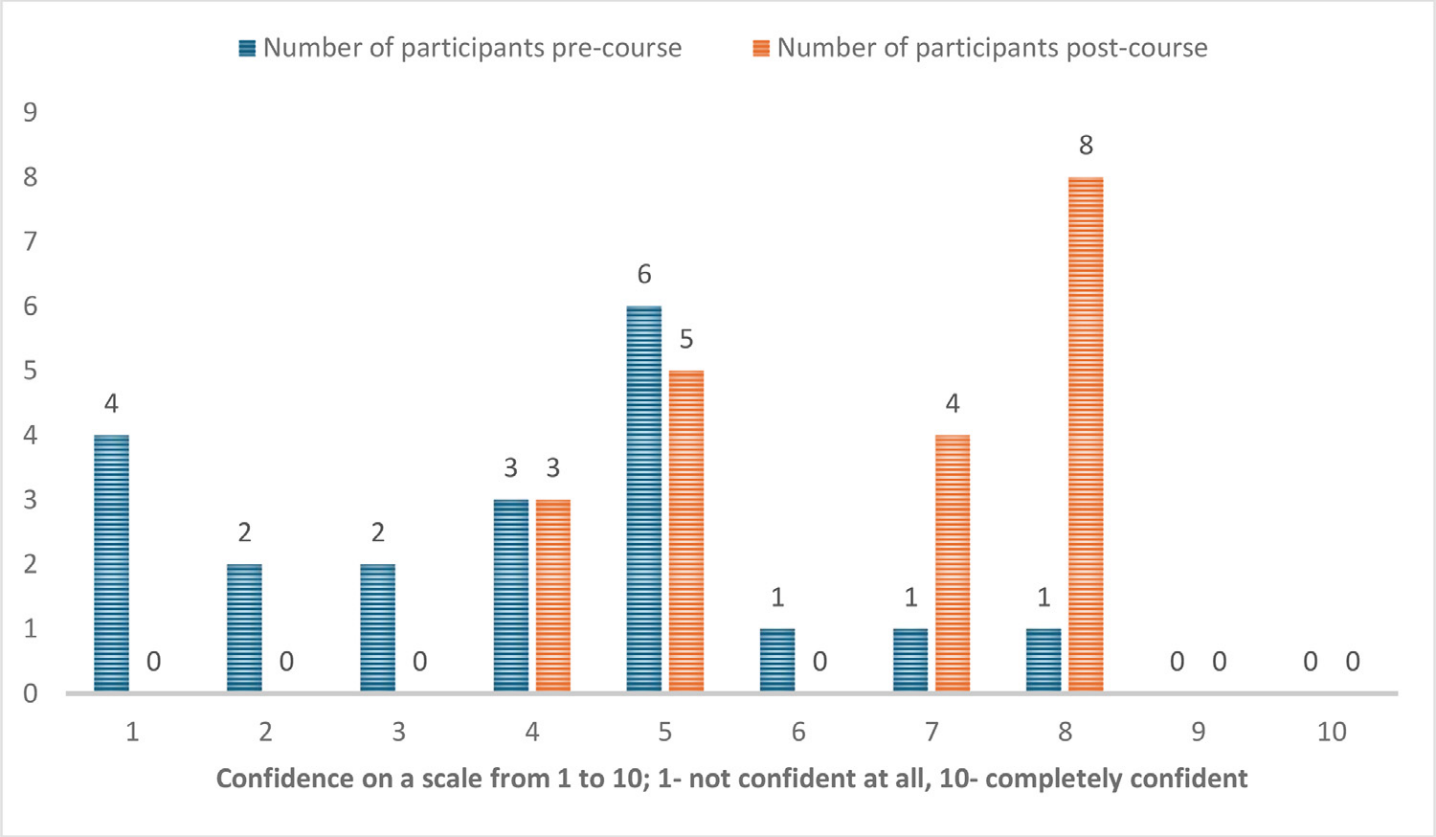
Self-reported confidence to identify the anatomical structures in the bronchial tree pre- and post-course.

Overall, 20 people participated in the course. Their training grade, previous training and experience are provided in Table 1. Self-reported confidence to perform bronchoscopy unsupervised increased significantly from 3 (mean; range 1–9) pre-course to 6 post-course (mean; range 1–9, both rounded to nearest number on scale of 1–10); *p* < 0.01 and self-reported confidence to identify endobronchial structures was also significantly increased from 4 to 6 (rounded to nearest number); *p* < 0.01 with the distribution as displayed in Fig. 1, Fig. 2.

**Table 1:** Candidates’ training grade, previous training and experience.

Training grade:	
Advanced critical care practitioner Junior fellow/pre-specialty training Core ICM or anaesthesia training Senior ICM or anaesthesia specialty training Specialty and associate specialist	3 7 2 6 2
Previous training in bronchoscopy	
None Ad hoc teaching in the clinical setting Specialised course	11 6 3
Number of bronchoscopies performed pre-course	
None <5 5–10 10–20 >20	9 6 2 2 1

**Fig. 3:**
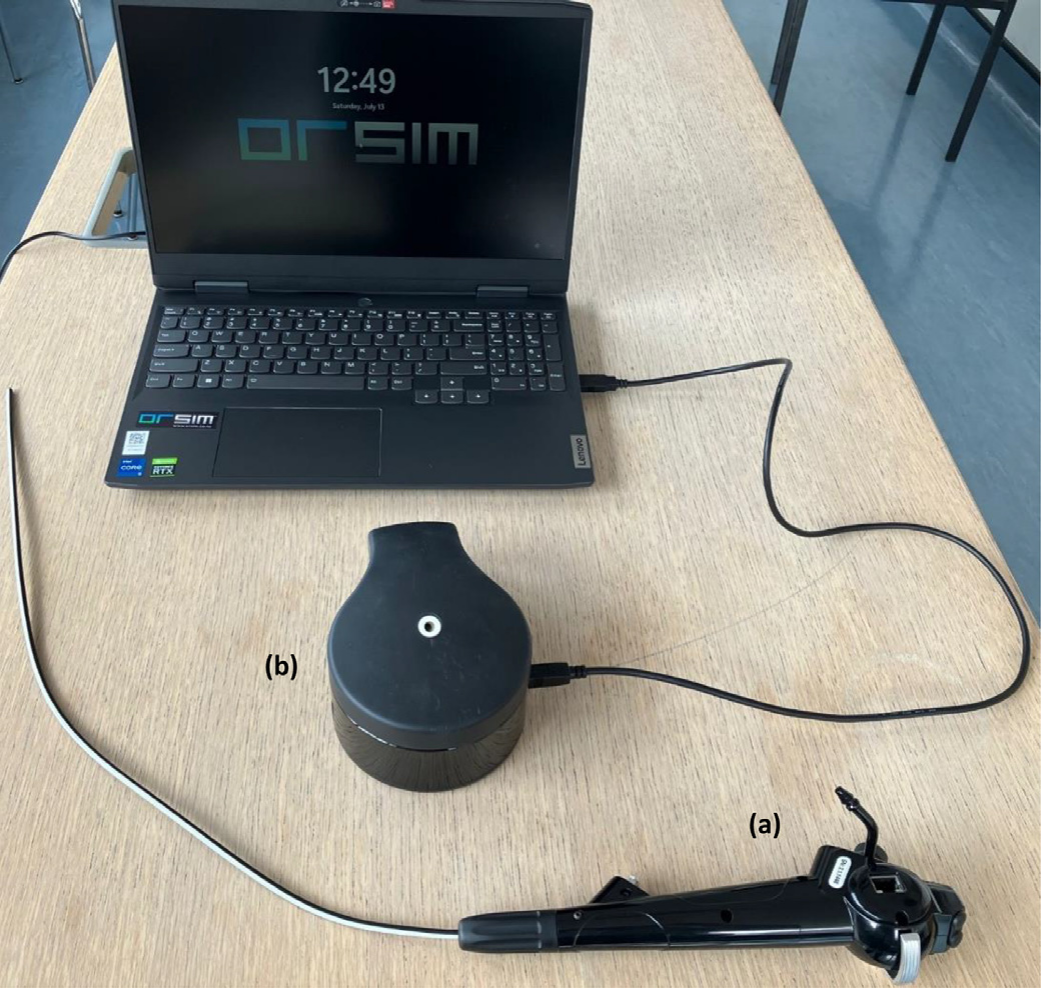
ORSIM bronchoscopy simulator consisting of a realistic handheld bronchoscope body (a), interface to feed distal end of bronchoscope through (b) and laptop, which can in turn be attached to a projector.

**Fig. 4:**
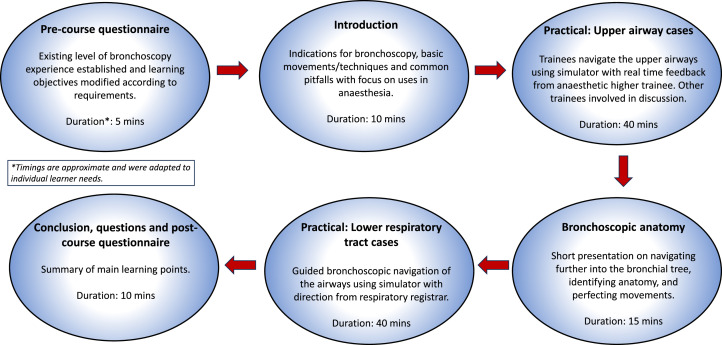
A flow diagram to demonstrate an overview of the structure of the bronchoscopy simulation session.

**Fig. 5:**
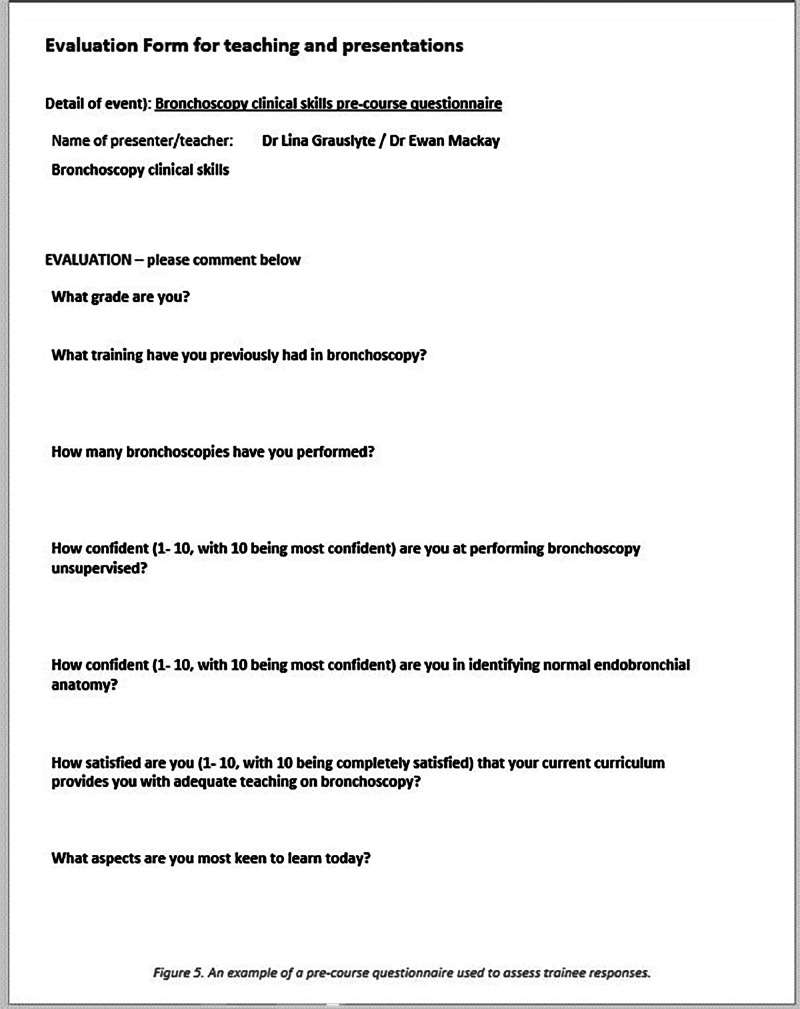
Example of pre-course questionnaire.

The self-reported confidence to perform bronchoscopy correlated moderately with the candidates’ grade (Pearson's correlation coefficient 0.38) and strongly with how many bronchoscopies they had performed in the past (Pearson's correlation coefficient 0.83). All course candidates who were in training (*n* = 8, ICM – intensive care medicine or anaesthetics) reported their satisfaction with their curriculum in terms of providing adequate teaching on bronchoscopy at 3 or lower (on a scale from 1 to 10).

We asked all course participants what they were keen to learn on the day of the course. One of the two main themes that emerged in the answers were anatomy (*n* = 11, with comments from trainees, such as ‘learn to navigate the bronchial tree’, ‘refresher in anatomy’, ‘navigate the anatomy’, ‘differentiate normal versus abnormal anatomy’). The second most often mentioned learning focus was based around increasing familiarisation with basic control of the bronchoscope (*n* = 10, ‘to get better at manipulating the bronchoscope‘, ‘different approaches to bronchoscope handling’, ‘familiarisation with the bronchoscope’). All the answers focused on learning the basics.

All course participants rated the usefulness of the course as 9 or 10 out of 10 and the main suggestion for the future was more opportunity to train in bronchoscopy (‘would love regular training’, ‘regular practice would be great’, ‘would like more sessions’, ‘more sessions’).

## Discussion

Bronchoscopy is an essential procedure that can be performed by doctors practising in various specialties (respiratory, thoracic surgery, intensive care).^11^ It is a procedure used both for diagnosis (detection of lung cancers, airway and lung injury from heat/fire/chemicals, cause of haemoptysis, bronchial washings to aid in diagnosis) and for treatment (foreign body removal, brachytherapy, bronchial washing, endobronchial lung volume reduction).^12^ Traditionally bronchoscopy teaching in all clinical settings took place using real patients. The ‘see one, do one, teach one’ approach, even though tested by time, poses significant risks in the case of bronchoscopy training, such as procedural anxiety for the operator, inappropriate diagnostic and/or therapeutic techniques, peri- and post-procedural complications^13^ and irregular training opportunities, dependent on the caseload. Although numbers of intensive care trainees in our sample was low, it is of significance that all appeared dissatisfied with their current curriculum, scoring their satisfaction a 3 or less out of 10.

Bronchoscopy training is more formalised in other specialties such as respiratory or cardiothoracic surgery and literature has looked at how to optimise training pathways within these specialties.^9^ However, staff working in intensive care are also expected to perform bronchoscopy to an acceptable basic standard, but this training pathway is less formal with less literature on the subject.^1^^,^^2^ Beyond sporadic bedside teaching, there are limited opportunities for formal training in this field for trainees within this specialty, which was reflected with the reported dissatisfaction with access to bronchoscopy training in our sample.

Achieving competence in a procedure is a continuous process and is likely variable between individuals. British Thoracic Society (BTS) guidelines acknowledge this, but have previously suggested that 50 bronchoscopies should be performed under direct supervision and 50 under indirect supervision to achieve competence.^14^ This is mirrored in respiratory deaneries in the UK requiring 100 flexible bronchoscopies for certificate of completion of training (CCT). For interventional procedures in already competent bronchoscopists, several respiratory societies and professional bodies have empirically suggested performing 20 bronchoscopies as an adequate number to achieve competence.^15^^,^^16^ Mahmood *et al* have found that, for novices, the learning curves to achieve competence in bronchoscopy vary widely^17^ and this is reflected in the Faculty of Intensive Care Medicine (FICM) guidelines for UK trainees,^18^ which do not provide a set number of procedures for competence but recommend an individualised approach with DOPS (direct observed procedural/practical skills) assessments and a recorded logbook. Among our study participants, only one reported having performed more than 20 bronchoscopies and two noted having done anywhere between 10–20, with the majority having performed up to 10 bronchoscopies.

Often, lack of exposure to bronchoscopy training results from clinical circumstances (lack of time, lack of patients in intensive care units requiring bronchoscopy, reluctance due to lack of experience).^12^^,^^19^ A possible way to address this is using bronchoscopy simulators and manikins. Vieira *et al* recently published a review of possible training tools for learning bronchoscopy, with the authors concluding that currently there are a variety of tools that can successfully and reliably complement regular methods of bronchoscopy training.^20^ Numerous static models for practising bronchoscopy have been cited in the literature, from simple low-cost models (not available commercially) created from corrugated ventilator tubing^21^ to moulded commercially available anatomical models such as the Air Sim Bronchi X (Trucorp; Craigavon, Ireland) / Bronchoscopy Training Model (Adam Rouilly; Kent, UK), which are more anatomically accurate but also expensive. A separate bronchoscope is required for these models and they suffer from the same disadvantages of lacking fidelity in terms of feel and their static nature (not changing with respiration), which limits realism.^20^

Garner *et al* sought to remedy this with a model based on preserved porcine lungs attached to bellows to mimic respiration, and operators compared it favourably in terms of feel and fidelity.^22^ However, porcine lungs differ anatomically from human lungs and this makes learning the anatomy of bronchoscopy difficult from this model alone, and the longevity of such models is not known.

Web-based interactive bronchoscopy simulations are available^23^ to map the bronchial tree and are useful adjuncts to aiding the identification of anatomical structures. However, different entry points are not available and it does not develop the muscle memory of navigating into bronchopulmonary segments and is limited in this regard. Given the limitations of static models and simulations displaying anatomy, sessions using these as teaching aids are likely to require more input from trained personnel to lead sessions and critique technique.

High-fidelity simulators include the Accutouch Endoscopy Simulator (Immersion Medical; Gaithersburg, USA) and the ORSIM (used here) which have considerable cost, potentially making them less accessible. The ORSIM simulator has many advantages; it is reasonably similar to a real bronchoscope in weight and feel, it is portable and different entry points can be used for practice. It has more of a focus on upper airway emergencies with the basic package, although additional bronchoscopy modules with lower respiratory tract pathology and questions are available. However, drawbacks are cost, and the scope does not have any channels through which biopsies can be practised. Similarly, the scenarios do not cover important aspects such as pre-medication, which is an important part of bronchoscopy. Useful adjuncts for learning associated practical aspects such as pre-medication and aftercare (in addition to national guidelines and published material) include case-based learning materials^24^ that can help to provide more of an overview of bronchoscopy.

If simulators are used, additional logistical considerations that need to be addressed are how and when the trainees receive the teaching. When bronchoscopy is taught in the clinical setting, learning takes place during clinical hours and form part of their clinical duties. When Cold *et al* rolled out a bronchoscopy simulation course for their study, one of the reported reasons for trainees not completing the course was lack of time or being ‘too busy’.^25^ Trainees in our pilot were keen to suggest that they were looking for more learning opportunities to perform flexible bronchoscopy, and this would appear to therefore not pose a significant threat to engagement. For this to be effective, it is likely that time out of clinical duties for study leave may be required.

A major limitation of this quality improvement project was that our team only assessed self-reported confidence and did not assess pre- and post-course competence, which is more difficult to define. It is hoped that greater knowledge of anatomy and confidence might correlate to some degree with procedural competence, but this cannot be assumed. The authors did consider additional quantitative measures to assess competence, such as timing simulated scenarios or recording the number of collisions with the bronchial walls, but it is unclear if this would differentiate competence in bronchoscopy from mastery of the simulator itself. Formative assessments of competence from independent operators may be of use, but it is important to maintain psychological safety of participants and an awareness of being assessed may alter the dynamic of the teaching session.

Nonetheless, other measures of competence such as the Bronchoscopy Skills and Tasks Assessment Tool (BSTAT) and Bronchoscopy Step-by-Step Evaluation Tool (BSET) may represent a more robust measure of competence for future studies.^26^^,^^27^ Additionally, we did not follow up at longitudinal checkpoints with our study participants to assess self-reported impact on their clinical practice following the course, which limits longitudinal extrapolation of our results. It also needs to be noted that our candidates, although working in the intensive care setting, came from a wide variety of backgrounds and a large variability in previous experience with bronchoscopy could have impacted perception of how beneficial the training session was. Nonetheless, our project is useful in identifying trainee dissatisfaction with current training and in putting forward a potential adjunct to training. To investigate further we are planning to expand our course and include analysis at different time-points to investigate if the changes are sustained.

Another aspect of bronchoscopy training that is worth considering is the circumstances in which most bronchoscopies are performed in the intensive care setting. Frequently, decision to perform bronchoscopy and preparing appropriately is as important as the bronchoscopy itself. These aspects cannot be simulated using a simulator and require additional teaching either in the clinical setting or in the form of pre-course learning materials for self-guided learning.

## Conclusions

Bronchoscopy, performed at a basic level, is an essential tool for an intensive care doctor. Our quality improvement pilot, although limited in numbers, certainly suggested that intensive care trainees have limited opportunities to practise flexible bronchoscopy, which may well have been exacerbated during the COVID pandemic. During COVID, education had to evolve due to social distancing, PPE and other physical constraints. Based on the significant increase in self-reported confidence and positive feedback from the course participants, short courses using simulators could potentially assist in ensuring the required teaching of bronchoscopy. This also adds to the current literature that suggests that simulators may be of great value in increasing exposure to this clinical skill, in a safe environment. Further studies are needed to assess how this exposure translates into clinical competence and the potential longevity of change.

## CRediT authorship contribution statement

**Ewan Christopher Mackay:** Writing – review & editing, Writing – original draft, Methodology, Formal analysis, Data curation, Conceptualization. **Lina Grauslyte:** Writing – review & editing, Writing – original draft, Methodology, Formal analysis, Data curation, Conceptualization.

## Declaration of competing interest

The authors declare that they have no known competing financial interests or personal relationships that could have appeared to influence the work reported in this paper.
